# Cell Adhesion Molecules as Modulators of the Epidermal Growth Factor Receptor

**DOI:** 10.3390/cells13221919

**Published:** 2024-11-19

**Authors:** Irina Kozlova, Vladimir Sytnyk

**Affiliations:** School of Biotechnology and Biomolecular Sciences, The University of New South Wales, Sydney, NSW 2052, Australia; i.kozlova@unsw.edu.au

**Keywords:** cell adhesion, cell proliferation, apoptosis, cancer, integrins, cadherins, immunoglobulin superfamily of cell adhesion molecules

## Abstract

Cell adhesion molecules (CAMs) are cell surface glycoproteins mediating interactions of cells with other cells and the extracellular matrix. By mediating the adhesion and modulating activity of other plasma membrane proteins, CAMs are involved in regulating a multitude of cellular processes, including growth, proliferation, migration, and survival of cells. In this review, we present evidence showing that various CAMs interact with the epidermal growth factor receptor (EGFR), a receptor tyrosine kinase inducing pro-proliferative and anti-apoptotic intracellular signaling in response to binding to several soluble ligands, including the epidermal growth factor. We discuss that CAMs are involved in regulating EGFR signaling by either potentiating or inhibiting the soluble ligand-dependent activation of EGFR. In addition, CAMs induce soluble ligand-independent forms of EGFR activity and regulate the levels of EGFR and its ligand-induced degradation. The CAM-dependent modulation of EGFR activity plays a key role in regulating the growth, proliferation, and survival of cells. Future research is needed to determine whether these processes can be targeted in both normal and cancerous cells by regulating interactions of EGFR with various CAMs.

## 1. Introduction

Cell adhesion molecules (CAMs) are cell surface glycoproteins that mediate cell-to-cell or cell-to-extracellular-matrix adhesion. They form adhesive bonds through either the homophilic binding of their extracellular domains to the extracellular domains of the same molecules in the plasma membrane of adjacent cells or through the heterophilic binding of their extracellular domains to other molecules in the plasma membrane of adjacent cells and in the extracellular matrix. In addition to mechanical stabilization of the contacts between a cell and its environment, CAMs determine the specificity of cell-to-cell interactions. CAMs are also involved in regulating many other cellular processes, including cell proliferation, growth, migration, and differentiation, by inducing intracellular signaling cascades comprising changes in the levels of various second messengers and activities of many enzymes and resulting in remodeling of the cytoskeleton, changes in gene expression, protein synthesis, and trafficking, and other processes [1,2,3,4]. CAMs are not enzymes and induce intracellular signaling cascades by interacting with other proteins at the plasma membrane. In this review, we summarize current literature demonstrating that CAMs modulate activity and signal through the epidermal growth factor receptor (EGFR), a receptor tyrosine kinase involved in regulation of cell proliferation, migration, survival, and differentiation [5,6].

## 2. Major Families of CAMs

**Figure 1:**
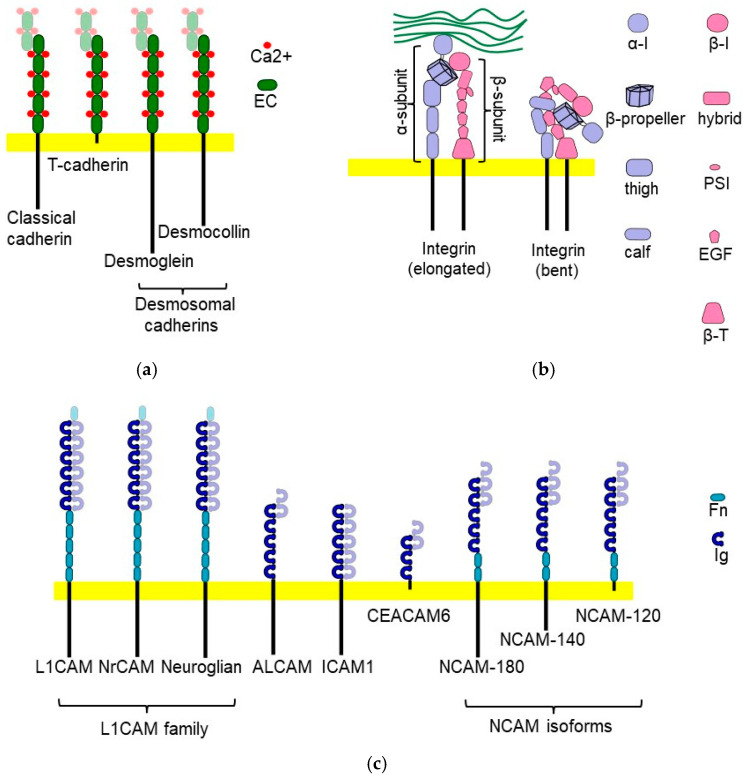
Schematic diagram showing examples of CAMs of major superfamilies. (**a**) Cadherin family members mentioned in the main text, including classical cadherins (E-, N-, P-cadherins), GPI-anchored T-cadherins, and desmosomal cadherins (desmogleins and desmocollins). Extracellular domains of cadherins are characterized by the presence of EC modules. Ca^2+^ ions bind between EC modules, stabilizing elongated conformation of the cadherin ectodomain. The membrane distal EC module of cadherins homophilically interacts in *trans* with the membrane distal EC module of cadherins on the neighboring cell. (**b**) Integrins are composed of α and β subunits. The extracellular domain of the α subunit contains the seven-bladed β propeller, thigh module, and two calf modules. The α-I module can be inserted between blade two and three of the β propeller. The extracellular domain of the β subunit contains the β-I module inserted in a hybrid module inserted in the plexin-semaphorin-integrin (PSI) domain. This is followed by four EGF repeats and β-T unit. Active elongated (left) but not bent (right) integrins bind to the extracellular matrix ligands. (**c**) IgSF members mentioned in the main text, including members of the L1CAM family and major NCAM isoforms. The extracellular domains of IgSF members are characterized by the presence of Ig modules and may also contain FnIII modules. IgSF members are either transmembrane or GPI-anchored proteins. They can homophilically interact in *trans* with the extracellular domains of CAMs on adjacent membranes via immunoglobulin domains.

Members of several major families of CAMs, including cadherins, integrins, and the immunoglobulin superfamily, have been implicated in regulating EGFR (Figure 1). These CAMs, grouped into superfamilies based on the presence of certain structural domains [7,8], are reviewed only briefly below, and references to other more in-depth reviews on the structure and functions of these CAMs are provided for further reading.

Cadherins are plasma membrane localized proteins that form several subfamilies, including type I and type II cadherins (collectively referred to as classical cadherins), desmosomal cadherins, protocadherins, and atypical cadherins [8,9,10] (Figure 1a). Members of this superfamily are characterized by the presence of several approximately 100-amino-acid-long extracellular cadherin (EC) domains [11]. Binding of calcium ions between EC domains is required for a molecule to acquire an elongated shape enabling formation of a homophilic adhesive bond in *trans* with a cadherin molecule on the cell surface of another cell [12]. Classical and desmosomal cadherins are proposed to interact in *trans* via swapping the β strands on the membrane distal EC1 domains or by forming X-dimers via extensive surface interactions between the two membrane distal EC1 and EC2 domains of each cadherin molecule [13,14]. Studies using truncated cadherin proteins showed that other EC repeats also contribute to homophilic interactions. For example, the full-length extracellular domain of Xenopus C-cadherin, comprising five EC1-EC5 domains, has a much higher binding affinity compared to a truncated extracellular cadherin domain comprising only EC1 and EC2 repeats [15]. Cadherin molecules in the plasma membrane of the same cell can also homophilically interact with each other in *cis*. These interactions are mediated by the EC1 domain of one molecule and EC2 domain of the neighboring cadherin molecule [16,17]. Homophilic interactions of cadherins in *cis* and *trans* are highly mutually cooperative, prolonging the lifetime of the adhesive bonds and resulting in strong adhesion [18]. The intracellular domains of cadherins are linked to the cytoskeleton. The intracellular domains of classical cadherins are connected to the cytoskeleton through β catenin [8], while intracellular domains of desmosomal cadherins interact with plakoglobin that anchors them to intermediate filaments, leading to the formation of desmosomes [19]. In addition to mechanically anchoring cells together, cadherins regulate various cellular processes, such as proliferation, apoptosis, and cellular differentiation, by inducing intracellular signaling [9].

Integrins are heterodimers made of non-covalently bound α and β subunits (Figure 1b). In vertebrates, there are eight different β subunits and eighteen α subunits, which can produce twenty-four different dimer combinations [8,20,21]. Each subunit consists of a large extracellular domain, transmembrane domain, and short intracellular domain. The extracellular domain of the α subunit is composed of a seven-bladed β propeller, a thigh module, and two calf modules. Nine of the eighteen integrin α subunits have an additional α-I module inserted between blades two and three of the β propeller. The extracellular domain of the β subunit comprises a β-I module inserted in a hybrid module inserted in the plexin-semaphorin-integrin domain followed by four cysteine-rich epidermal growth factor (EGF) modules and a β tail. Cytoplasmic domains of α and β subunits are approximately 1000 and 750 amino acids long, respectively [22]. Integrins play a central role in adhering cells to the extracellular matrix (ECM) ligands recognized by their extracellular domains and in transmitting signals from the ECM to the cytoplasm via their intracellular domains that bind the cytoskeleton and signaling proteins [23]. The binding to the ECM ligands is regulated by conformational changes in integrins. Integrins in the bent conformation exhibit low affinity for extracellular ligands, while integrin activation via transition to an extended conformation results in a significant increase in the binding affinity [23,24]. Activation of integrins can be triggered by the external forces from the ECM and by the interaction of their intracellular domains with a cytoskeletal protein talin [24,25].

Members of the immunoglobulin superfamily (IgSF) are characterized by the presence of the immunoglobulin-like (Ig) modules in their extracellular domains [8,26] (Figure 1c). In addition to the Ig modules, the extracellular domains of these molecules often contain additional modules, such as fibronectin type III (FnIII) repeats [4,8,27]. The IgSF is further subdivided into families comprising molecules featuring similar structural organization of their extracellular domains, including but not limited to the neural cell adhesion molecule (NCAM) family, L1CAM family, IgLON family, Nectins and Nectin-like protein family, and other families [8,26,27,28]. Most IgSF members are transmembrane proteins. However, this superfamily also contains the largest number of members attached to the plasma membrane by the glycosylphosphatidylinositol (GPI) anchor and lacking transmembrane and cytoplasmic domains [27]. Extracellular domains of many IgSF members can form homophilic adhesive bonds by interacting with the same molecules on the plasma membranes of adjacent cells. In addition, IgSF members heterophilically interact with other cell adhesion molecules of the IgSF and other families [26,29,30,31]. IgSF cell adhesion molecules transmit signals to the cytoplasm in response to their homo- and heterophilic interactions at the cell surface by interacting with the cytoskeleton and various signaling molecules through their intracellular domains [1,2,4,32].

In addition to integrins, cadherins, and IgSF members, other cell surface receptors mediating cell-to-cell and cell-to-ECM adhesion were implicated in regulating EGFR activity. While these receptors are not the focus of the present review, several examples are given in the text below.

## 3. Epidermal Growth Factor Receptor

**Figure 2:**
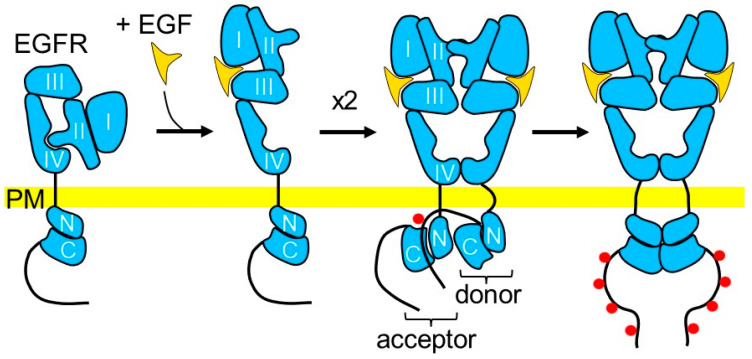
Schematic diagram of the ligand-dependent EGFR activation. In a ligand-free state, the interaction of domain II with domain IV masks the dimerization arm within domain II. Binding of EGF or other soluble ligands (not depicted) to domains I and III leads to a conformational change exposing the dimerization arm and leading to the dimer formation. Within the dimer, the C-lobe (C) of the kinase domain of the donor receptor binds to the N-lobe (N) of the kinase domain of the acceptor receptor, allowing insertion of the carboxy-terminal tail of the donor into the active site of the kinase domain of the acceptor, resulting in the kinase activation and autophosphorylation (red circles). PM—plasma membrane.

Epidermal growth factor receptor (EGFR) is a member of the ErbB family of receptor tyrosine kinases, which also includes HER2 (erbB2), HER3 (erbB3), and HER4 (erbB4). EGFR is composed of the large extracellular domain, short transmembrane region, followed by the intracellular juxtamembrane region, tyrosine kinase domain, and carboxy-terminal tail containing multiple phosphorylation sites. The extracellular domain of EGFR consists of four subdomains (domains I, II, III, and IV, Figure 2). Subdomains I and III are ~37% identical and are involved in ligand binding. Subdomains II and IV are rich in cysteines and contain a string of disulfide bonds. In mammals, EGFR binds several ligands, including epidermal growth factor (EGF), transforming growth factor-alpha (TGFα), heparin-binding EGF-like growth factor (HB-EGF), amphiregulin (AREG), betacellulin (BTC), epiregulin (EREG), and epigen (EPGN) [33]. EGFR ligands are synthesized as transmembrane precursor proteins composed of the N-terminal extension, EGF module, juxtamembrane stalk, hydrophobic transmembrane domain, and C-terminal cytoplasmic tail. The functional EGF module is released into the extracellular space via the proteolytic cleavage of the precursor protein and can then interact with EGFR on distant or neighboring cells or cells of its origin [33].

The mechanisms of EGFR activation were reviewed previously [34,35] and are discussed here only briefly. The crystal structure analysis of the entire extracellular domain of the inactive EGFR revealed the intramolecular interaction between cysteine-rich subdomains II and IV, where a hairpin projecting from the domain II is inserted between similar (but shorter) loops of the domain IV [36]. Ligand binding to domain I induces rotation of domains I and II by 130° with respect to domains III and IV, which allows docking of the ligand to both domain I and domain III. The bivalent attachment of the ligand to domains I and III results in the disruption of the autoinhibitory interaction between domains II and IV, allowing the binding of the extracellular domain of EGFR to the extracellular domain of another EGFR molecule and leading to the formation of EGFR dimers [36,37]. Dimerization enables autophosphorylation of the receptor. The kinase domain of EGFR consists of the N-lobe and C-lobe with an ATP-binding site located between the two lobes (Figure 2). At resting conditions, the kinase domain is autoinhibited. Dimerization allows the N-lobe of the kinase domain of one receptor (acceptor) to interact with the C-lobe of the kinase domain of another receptor (donor). The carboxy-terminal tail of the donor inserts into the active site of the acceptor kinase and activates it, resulting in phosphorylation of the carboxy-terminal tail of the donor [37,38]. Activation of the EGFR kinase and autophosphorylation of EGFR can be triggered not only by ligand binding but also by an increase in the local concentration of the receptor or its kinase domain [39].

## 4. EGFR Signaling and Trafficking

Autophosphorylation of EGFR induced by ligand binding creates binding sites for other kinases and adaptor proteins on its intracellular domain [40] linking EGFR to various signaling pathways, which involve hundreds of proteins and protein interactions, leading to phosphorylation of thousands of proteins [41] and resulting in various cellular processes, such as cell proliferation and growth [36,38]. Among the EGFR-dependent signaling pathways, the Ras/Raf/mitogen-activated protein kinase (MAPK, also called Erk1/2) pathway and PI3K/Akt/mTOR pathway are probably most well-characterized [5,6]. The signaling pathways initiated by EGFR vary depending on the cell type and ligands EGFR binds to. For example, EGF, TGF, HB-EGF, and BTC similarly potently activate the MAPK pathway in HeLa cells, while the PI3K-Akt pathway is most potently activated by HB-EGF, less potently activated by BTC and EGF, and only weakly activated by high concentrations of TGFα [42]. The ligand-induced EGFR dimerization triggers internalization of the receptor [43]. In fibroblasts, inhibition of EGFR endocytosis by dynamin depletion does not affect the activation of the MAPK and Akt signaling, indicating that these responses can be primarily mediated by activated EGFR located in the plasma membrane [44]. EGFR remains active in early endosomes after internalization. However, the cell surface and internalized EGFR are bound to distinct binding partners, suggesting that they regulate different cellular pathways [45].

EGFR is internalized via clathrin-dependent and independent mechanisms. In various cell types, low concentrations of EGF trigger redistribution of EGFR to clathrin-coated pits followed by EGFR internalization, predominantly via clathrin-mediated endocytosis [46,47,48]. However, at higher EGF concentrations, a large proportion of EGFR is sorted to caveolae for clathrin-independent internalization [49]. EGFR internalized via clathrin-mediated endocytosis is not targeted for degradation, but instead is recycled to the cell surface. By contrast, clathrin-independent internalization preferentially commits the receptor to degradation [50]. Intriguingly, EGFR can also regulate endocytosis. EGF induces activation of c-Src family kinases followed by clathrin heavy-chain phosphorylation, clathrin redistribution to the cell periphery, and EGF endocytosis [51]. In PANC-1 and NR6 cells, EGFR can also be internalized by EGF-induced actin-enriched dynamic protrusions called dorsal “waves” via a clathrin- and caveolae-independent mechanism [52].

Internalization of EGFR from the plasma membrane into endosomes plays a key role in the inactivation of EGFR-dependent signaling via mechanisms that were reviewed previously [53,54] and discussed here only briefly. Maturation of endosomes is accompanied by their movement towards the center of the cell, luminal acidification, enlargement of the vesicular subcompartments, and appearance of the intralumenal vesicles typical of multivesicular bodies. From here, receptors are either recycled back to the cell surface by recycling endosome or, if trapped in the intraluminal vesicles, stay in multivesicular bodies that mature into late lysosomes where EGFR is degraded [53,54]. The deactivation of the internalized receptors occurs before degradation most likely due to the ligand removal [45]. The trafficking of EGFR depends on the ligands it binds to. In HEp2 cells, all out of six tested EGFR ligands, including EGF, TGFα, HB-EGF, BTC, AREG, and EPGN, induce the targeting of EGFR to early endosomes. However, HB-EGF- and BTC-bound EGFR is targeted for lysosomal degradation, while EGF causes most but not all EGFRs to be degraded by lysosomes and allows remaining EGFR to be recycled back to the cell surface, and AREG, TGFα, and EPGN allow recycling of most EGFR back to the cell surface [55]. The lysosomal targeting of EGFR requires ubiquitination of the receptor. In PAE cells, a non-ubiquitinated EGFR mutant is internalized at a rate similar to non-mutated EGFR [56] but is not localized to the intraluminal vesicles for subsequent entry to the lysosomes and is instead recycled back to the plasma membrane [57]. EGFR is ubiquitinated by the Cbl E3 ligase and must be de-ubiquitinated in a proteasome-dependent manner prior to its lysosomal degradation [58].

Altogether, previous research indicates that soluble extracellular ligands of EGFR induce activation of EGFR at the cell surface, trigger EGFR-dependent signaling, and regulate EGFR sorting, determining its subsequent intracellular trafficking and inactivation. Below, we review the current evidence demonstrating that, in addition to the canonical pathways of EGFR activation by soluble ligands, the EGFR activity and levels are regulated in a ligand-independent manner by cell-surface-localized members of several families of CAMs, which also modulate the ligand-dependent activation of EGFR.

## 5. Regulation of EGFR by Cadherins

In early studies, classical cadherins were implicated in inhibition of the ligand-dependent EGFR activity. The EGF-dependent activation of EGFR is strongly inhibited in cultures of normal human breast epithelial cells when they reach apparent confluence coinciding with accumulation of EGFR and E-cadherin at contacts between cells. The EGF-dependent activation of EGFR is restored in cells treated with anti-E-cadherin antibodies, which disperse E-cadherin from the cell-to-cell contacts [59]. Later research showed that ligation of the cell surface E-cadherin in A431 cells using the purified and functionally active extracellular domain of E-cadherin results in inhibition of EGF-stimulated phosphorylation of EGFR at Tyr845, a site that is transphosphorylated by c-Src after it is recruited and activated by EGFR. E-cadherin ligation also blocks the STAT5-dependent signalling in these cells but does not affect the autophosphorylation of EGFR or ERK signalling [60].

**Figure 3:**
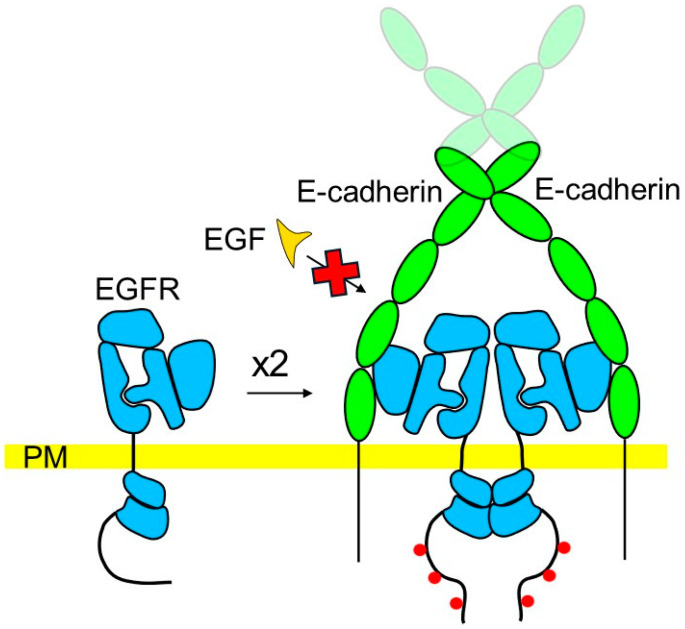
A hypothetical model of the ligand-independent EGFR activation at E-cadherin adhesive bonds. The extracellular domain of E-cadherin contains five extracellular cadherin modules (green). The first two N-terminal cadherin modules mediate formation of the cis-dimers by two E-cadherin molecules within the plasma membrane (PM) of the same cell. The first two N-terminal cadherin modules also mediate formation of adhesive bonds by binding to E-cadherin molecules (represented by only the first three cadherin modules for simplicity) in the plasma membrane of the adjacent cell. Binding of the extracellular domains of EGFR molecules to the extracellular domains of E-cadherins in adhesive bonds leads to clustering of EGFR, allowing its ligand-independent autophosphorylation. Interactions between EGFR and E-cadherins occlude binding sites for EGF and other ligands and block the ligand-mediated EGFR activation.

While classical cadherins inhibit the ligand-dependent activation of EGFR, a growing body of research indicates that cadherins also induce ligand-independent EGFR activation (Figure 3). In cultures of immortalized non-tumorigenic keratinocyte HaCat cells, the assembly of E-cadherin-mediated adherence junctions leads to the recruitment of EGFR to E-cadherin-containing complexes, ligand independent activation of EGFR, and stimulation of the MAPK signaling pathway [61]. A similar effect is found in multicellular aggregates formed by cultured HSC-2 squamous carcinoma cells, where EGFR is recruited to sites of E-cadherin-mediated adhesion between cells, forms a complex with E-cadherins, is activated in a ligand-independent manner, and induces ERK1/2 phosphorylation. The antibody-induced crosslinking of E-cadherins at the cell surface of these cells induces EGFR activation, while antibodies blocking E-cadherin homophilic trans interactions reduce EGFR activation in multicellular aggregates [62]. In MCF10A mammary epithelial cells, E-cadherin engagement induced by the calcium switch or clustering using specific antibodies also leads to formation of the E-cadherin/EGFR complex and EGFR autophosphorylation. Further analysis using cadherin-negative MDA-MB-435 mammary epithelial cells transfected with cadherins showed that EGFR binds to the extracellular domain of E-cadherin but does not interact with N-cadherin [63]. In E-cadherin-positive MDA-MB-231 human breast carcinoma cells, E-cadherin also forms a complex with EGFR. The E-cadherin function blocking antibody recognizing the extracellular domain of E-cadherin reduces the levels of autophosphorylated EGFR in these cells, indicating that E-cadherin promotes EGFR activation [64]. Ablation of E-cadherin expression in Madin-Darby canine kidney (MDCK) epithelial cells reduces levels of phosphorylated EGFR, an effect which is reversed by re-expression of E-cadherin [65]. Further confirming the role of the extracellular domain of E-cadherin in EGFR activation, the soluble extracellular domain of E-cadherin binds to EGFR at the cell surface of MDCK cells and induces EGFR phosphorylation, recruitment of Grb2 to EGFR, and subsequent downstream activation of the ERK1/2 and Akt signaling pathways. In contrast to stimulation with EGF, stimulation with the extracellular domain of E-cadherin does not result in internalization of EGFR [66].

Non-classical cadherins are also involved in EGFR regulation. In A431 cutaneous squamous carcinoma cells, silencing of T-cadherin, a GPI-anchored cadherin, promotes the EGF-induced activation and internalization of EGFR and potentiates the EGF-induced increase in cell migration, while overexpression of T-cadherin reduces the response to EGF and induces EGFR accumulation in lipid rafts [67], indicating that T-cadherin inhibits the ligand-dependent EGFR activation. A desmosomal cadherin Desmoglein-1 promotes keratinocyte differentiation by suppressing the EGFR/MAPK signaling during epidermal differentiation [68]. Desmoglein-1 also suppresses the EGF-induced EGFR-dependent formation of invadopodia, actin-based protrusions formed by cancer cells to facilitate invasion and metastasis. This effect depends on the binding of the Desmoglein-1 intracellular domain to the ErbB2-binding protein Erbin [69].

Interestingly, EGF-induced invadopodium formation is suppressed by knock-down of another desmosomal cadherin, Desmoglein-2 [69], suggesting that in contrast to Desmoglein-1, it positively regulates EGFR. Desmoglein-2 interacts with EGFR via its extracellular domain [70]. In HaCaT keratinocytes, the loss of Desmoglein-2 leads to a reduction in the levels of EGFR and inhibition of the EGF-dependent EGFR autophosphorylation and STAT3 pathway activation but does not affect the EGF-induced MAPK signaling [71]. In A431 cells, overexpression of Desmoglein-2 increases cell migration in response to serum-containing medium, and this effect is blocked by an EGFR inhibitor indicating that Desmoglein-2 promotes EGFR-dependent cell migration [71]. Desmoglein-2 induces the ligand-independent c-Src-mediated activation of EGFR by dispersing c-Src and EGFR from the lipid rafts [71]. In enterocytes, the loss of Desmoglein-2 also results in a reduction in levels of EGFR and its c-Src-phosphorylated form in the intestinal epithelium [70]. Intriguingly, the interaction between Desmoglein-2 and EGFR is inhibited by EGF and the EGFR kinase inhibitor, suggesting that Desmoglein-2 competes with EGF for binding to EGFR and induces the ligand-independent EGFR activation, which strengthens the interaction between Desmoglein-2 and EGFR [70]. EGFR also co-immunoprecipitates with a desmosomal cadherin Desmocollin-2. The loss of Desmoglein-2 results in the suppression of the EGFR activation and MAPK pathway signaling and leads to a concomitant increase in Desmocollin-2 levels in SK-CO15 colon carcinoma cells. Overexpression of Desmocollin-2 suppresses the EGFR signaling in SK-CO15 cells, suggesting that Desmoglein-2 also regulates EGFR by modulating Desmocollin-2 levels [72].

## 6. Regulation of EGFR by Integrins

**Figure 4:**
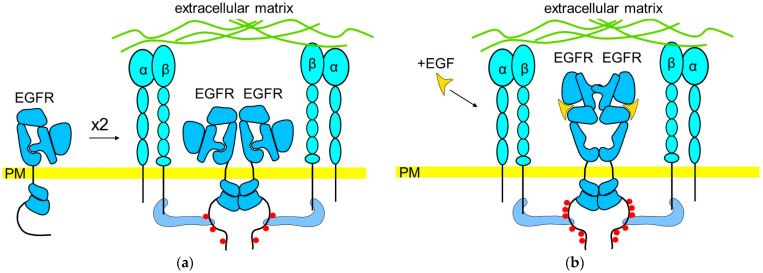
A hypothetical model of the integrin-mediated ligand-independent EGFR activation potentiating the ligand-dependent EGFR activation. (**a**) Dimers formed by α and β subunits of integrins cluster by binding to the extracellular matrix components (green). Binding of EGFR molecules to integrins via intracellular linker proteins (light blue) leads to clustering of EGFR molecules within the integrin-assembled scaffold, allowing partial ligand-independent autophosphorylation of EGFR. (**b**) Higher local concentration of EGFR within the integrin-assembled scaffold also potentiates the ligand-mediated EGFR activation. PM—plasma membrane.

Integrins induce EGFR activation in the absence of EGF in normal epithelial cells and in cancer cells (Figure 4a). Activation of integrins in N6 primary human skin fibroblasts, ECV304 endothelial cells, EGFR-transfected NIH-3T3 cells, and human keratinocyte-derived SCC12F2 cells using the extracellular matrix protein fibronectin or monoclonal antibodies against β1, αv, or β3 integrins induces transient EGFR autophosphorylation and activation of the MAPK and Akt signaling pathways [73,74,75]. Similarly, integrin-induced EGFR phosphorylation in response to attachment to fibronectin-, laminin-, or collagen I-coated plates is observed in Cos7, CV1, and several other different epithelial cells, including the prostate cell lines DU145 and RWPE-1, telomerase-immortalized retinal epithelial cells, A431, and primary prostate epithelial cells [76]. Inhibitors of EGFR block the integrin-induced activation of MAPK and PI3K/Akt pathways, indicating that integrins signal through EGFR [76]. Similarly, plating of A549 non-small cell lung cancer cells on an anti-β1 integrin activating antibodies results in EGFR phosphorylation even in the absence of EGF [77]. Interestingly, however, a collagen-binding α1β1 integrin inhibits EGFR phosphorylation through activation of T-cell protein tyrosine phosphatase (TCPTP), which is activated when it binds to the cytoplasmic domain of collagen-activated α1 integrin [78].

Growth factors induce only a transient activation of the MAPK signaling pathway in non-adhered suspended cells, while the integrin-mediated adhesion of cells is sufficient to sustain this signaling [79], suggesting that integrins promote the EGF-dependent activation of EGFR (Figure 4b). Indeed, integrins potentiate the EGF-dependent EGFR activation in various cells, including human foreskin fibroblasts [80], ECV304 endothelial cells [73,74], A549 non-small cell lung cancer cells, and the SK-MES1 lung squamous carcinoma cells [77]. Knock-down of β1 integrins in A549 cells does not affect the kinetics of EGFR signaling but rather decreases the extent of EGF-induced EGFR activation and phosphorylation of the downstream FAK, c-Src, Akt, and Erk1/2 [77]. EGFR forms a complex with β1 and αVβ3 integrins at the cell surface of ECV304 cells, EGFR-transfected NIH-3T3 fibroblasts, and human keratinocyte-derived SCC12 cells [73,74,75,81] and with α5β1 integrin in A2780 cells [82]. The formation of the complex is triggered by the binding of integrins to their ligands and coincides with EGFR phosphorylation, which requires the intracellular domain of integrins. The assembly of the EGFR/integrin complex depends on the activation of c-Src, which binds to integrins and phosphorylates EGFR and requires EGFR kinase activation [75,81]. EGF is not required for the binding of EGFR to integrins; however, the EGFR/integrin complex formation is enhanced in the presence of EGF [74]. In A2780 cells, the intracellular domains of α5 integrin and EGFR bind to Rab-coupling protein (RCP), and knock-down of RCP prevents co-immunoprecipitation of EGFR with α5β1 integrin [82], suggesting that EGFR does not bind to integrins directly but is rather connected to integrins via linker proteins such as RCP. Migration of A2780 cancer cells into matrigel depends on EGFR, since it is abolished after EGFR knock-down using siRNA. Inhibition of αvβ3 integrin with a soluble RGD ligand cilengitide promotes migration of A2780 cells into matrigel. This migration is dependent on engagement of α5β1 integrin with fibronectin and its binding to RCP, which links α5β1 integrin to EGFR. A truncated mutant of RCP that associates with α5β1 integrin but not with EGFR disrupts the cilengitide-induced migration of A2780 cells and EGFR activation, indicating that cell migration is promoted by α5β1 signaling through EGFR [82]. In human keratinocyte-derived SCC12 cells, the fibronectin-induced β1 integrin/EGFR complex formation is enhanced after depletion of ganglioside GM3 and inhibited after GM3 accumulation [75], indicating that the interactions of EGFR with integrins are modulated by lipids.

Integrins trigger EGFR phosphorylation on tyrosine residues 845, 1068, 1086, and 1173, but do not induce phosphorylation on tyrosine 1148, a major EGF-dependent autophosphorylation site [81]. The EGFR/integrin complex is disassembled within 30 min after integrin activation; however, EGFR phosphorylation is maintained, indicating that integrins trigger a sustained longer-lasting EGFR activation [75,81]. The integrin-dependent activation of EGFR in ECV304 cells is accompanied by an increase in EGFR cell surface levels, and this effect is blocked in cells with inhibited c-Src [81]. In wild-type HSC3 human oral squamous carcinoma cells, EGF-induced removal of EGFR from the cell surface is potentiated after the knock-down of β5 integrin, indicating that integrins also modulate the ligand-dependent endocytosis of EGFR [83]. In A2780 cells, cyclic RGD peptides that bind to and inhibit αvβ3 and αvβ5 integrins promote recycling of α5β1 integrins and EGFR to the cell surface but do not affect internalization of EGFR. The downregulation of α5 integrin using RNAi severely impairs EGFR recycling and EGF-driven EGFR autophosphorylation and induction of Akt signaling, indicating that they depend on α5β1 integrins in these cells [82]. However, the knock-down of β1 integrins in A549 cells leads to an increase in the cell surface expression of EGFR in the absence of EGF but does not affect the EGF-induced EGFR internalization and recycling [77], suggesting that integrins regulate EGFR trafficking in a cell-type-specific manner. Interestingly, EGF induces β1 integrin internalization in human cutaneous squamous carcinoma HSC-1 cells, and this effect is suppressed by T-cadherin, which reduces the activation of EGFR and inhibits caveolae-mediated endocytosis [84].

## 7. Regulation of EGFR by IgSF Members

Members of the IgSF are involved in regulating both ligand-dependent and ligand-independent activity of EGFR. Members of the L1CAM family of the IgSF promote ligand-independent EGFR activity. Human EGFR expressed in *Drosophila* Schneider 2 (S2) cells is activated in the absence of EGF at contacts established by cells co-expressing human L1CAM but not at contacts between non-transfected cells or cells expressing *Drosophila* cell adhesion molecule Fasciclin 1, indicating that L1CAM induces ligand-independent activation of EGFR [85,86]. Human L1CAM and human EGFR co-immunoprecipitate with each other when both proteins are expressed in S2 cells [85] or in human embryonic kidney HEK293 cells [87]. Interestingly, S2 cell aggregation assays show that human L1CAM can bind in *trans* to EGFR on membranes of other cells; however, this type of interaction is not sufficient to induce EGFR activation, indicating that interactions of L1CAM and EGFR in *cis* in the plasma membrane of the same cell are needed to induce EGFR activation [85].

In thyroid cancer K1 cells, EGFR co-immunoprecipitates with neural glia-related cell adhesion molecule (NrCAM), also a member of the L1CAM family [88]. The protein complex also contains α4β1 integrins, which most likely bind to the third Fn-like domain of NrCAM since antibodies against this domain suppress the MAPK and Akt signaling pathways in thyroid cancer FTC133 cells [88]. Proteolytic cleavage of NrCAM at the cell surface of K1 cells results in the release of the extracellular domain of NrCAM, which activates EGFR signaling when presented in the culture medium to thyroid cancer IHH4 cells, suggesting that it binds in trans to EGFR at the cell surface [88].

Functional studies in *Drosophila* suggest that the interactions between EGFR and L1CAM family members are evolutionary conserved. The loss of neuroglian, a *Drosophila* invertebrate homologue of L1CAM proteins, results in axonal pathfinding defects in the ocellar sensory system. These defects can be partially rescued by the gain of function of *Drosophila* EGFR, indicating that neuroglian signals through EGFR to guide axonal growth [89]. Overexpression of neuroglian or its GPI-anchored extracellular domain in wing sensory neurons and epithelial layers results in a reduction of wing size and the appearance of ectopic neuronal cells and severe axonal pathfinding defects, which can be rescued by decreasing doses of *Drosophila* EGFR, further indicating that neuroglian activates EGFR to regulate axonal growth [85].

Knock-down of activated leukocyte cell adhesion molecule (ALCAM) results in a reduction in EGFR phosphorylation and MAPK activation in nasopharyngeal carcinoma CNE-2R cells in the absence of EGF, suggesting that ALCAM also maintains the ligand-independent activity of EGFR [90]. However, ALCAM negatively regulates the ligand-dependent activation of EGFR. EGFR forms a complex with ALCAM in CNE-2R cells [90] and primary multiple myeloma cells and RPMI8226 cells [91]. In primary multiple myeloma cells and RPMI8226 cells, ALCAM associates with inactive ligand-free EGFR. ALCAM and EGF compete for binding to EGFR since ALCAM attenuates the binding of EGF to EGFR, while EGF disrupts the binding of EGFR to ALCAM [91]. Knock-down of ALCAM in RPMI8226 cells results in potentiation of EGFR phosphorylation and MAPK and Akt signaling in response to recombinant EGF or EGF secreted by bone marrow stem cells [91].

In contrast to ALCAM and L1CAM family members, the intercellular adhesion molecule-1 (ICAM-1) does not induce the ligand-independent EGFR activation but rather promotes the binding of EGF to EGFR and potentiates the ligand-dependent activation of EGFR [92]. Domain III of EGFR directly binds to the first N-terminal domain of ICAM-1. In triple-negative breast cancer cells, ablation of ICAM-1 expression suppresses while overexpression of ICAM-1 increases dimerization and phosphorylation of EGFR and activation of the EGFR-dependent signaling [92].

In oral squamous cell carcinoma, overexpression of carcinoembryonic antigen-related cell adhesion molecule 6 (CEACAM6) also potentiates the EGF-induced EGFR phosphorylation, activation of the MAPK and Akt signaling pathways, and cell migration [93]. EGF triggers binding of EGFR to CEACAM6 in the plasma membrane of these cells. The binding of CEACAM6 to EGFR depends on the complex N-glycosylation of CEACAM6. The inhibition of CEACAM6 glycosylation strongly attenuates its binding to EGFR and reduces the EGF-dependent activation of MAPK and Akt signaling [93].

IgSF family members were also involved in regulating EGFR degradation. The longest isoform of the neural cell adhesion molecule (NCAM) with the molecular weight 180 kDa (NCAM180) reduces basal levels of EGFR co-expressed with NCAM180 in transfected HEK 293 cells. NCAM180 also promotes EGF-triggered EGFR degradation by increasing the activity of the c-Cbl E3 ligase and, consequently, EGFR ubiquitination [94]. In rat cerebellar granular neurons, homophilic adhesion mediated by NCAM180 reduces EGFR levels and suppresses EGFR signaling to promote neurite outgrowth [94]. The extracellular domain of NCAM180 does not bind to EGFR, and the smaller isoform of NCAM with the molecular weight 140 kDa (NCAM140), which has an identical extracellular domain but shorter intracellular domain, does not regulate EGFR. Furthermore, overexpression of the dominant negative cytoplasmic intracellular domain of NCAM180 suppresses the NCAM180-mediated downregulation of EGFR levels, indicating that NCAM180 regulates EGFR via its intracellular interaction partners [94]. Interestingly, the interactions of NCAM with EGFR appear to have undergone changes during evolution, since overexpression of human NCAM140 causes axonal pathfinding defects in wing sensory neurons of *Drosophila*. These defects are suppressed by a 50% reduction in *Drosophila* EGFR levels, indicating that human NCAM140 can signal through *Drosophila* EGFR [95], although it does not activate mouse EGFR.

## 8. Regulation of EGFR by Other CAMs

Epithelial cell adhesion molecule (EpCAM) does not belong to the major superfamilies and mediates Ca^2+^-independent homophilic adhesion between epithelial cells [96]. The extracellular domain of EpCAM contains two epidermal growth factor (EGF)-like modules. Recombinant extracellular domain of EpCAM induces EGFR phosphorylation and MAPK and Akt activation in the absence of EGF in colon cancer HCT116, Colo205 and HT-29 cells [97] and in mesenchymal stem cells [98]. EGFR activation induced by the extracellular domain of EpCAM promotes cell migration of the colon cancer cells and this effect is abolished in cells treated with an EGFR inhibitor [97].

Ninjurin2, also known as nerve injury-induced protein 2, and its homologue ninjurin1 are homophilic cell adhesion molecules with no significant homology to any other known proteins [99]. In HT-29 human colon adenocarcinoma cells, primary human colon cancer cells, A172 human glioma cell line and primary human glioma cells, Ninjurin 2 forms a complex with EGFR [100,101]. In HT-29 and A172 cells, knock-down of Ninjurin2 leads to downregulation of the MAPK and Akt signaling, while overexpression of Ninjurin2 results in activation of MAPK and Akt in the absence of EGF, suggesting that Ninjurin2 induces the ligand-independent activation of EGFR [100,101].

CD44 proteins form a ubiquitously expressed family of CAMs that bind to the extracellular matrix components and mediate cell-to-cell and cell-to-extracellular-matrix interactions [102]. In HT1080 and U87MG cancer cells, the CD44s isoform of CD44 predominantly expressed in these cells interacts with Rab7A in the late-endosome/lysosome compartments, reduces levels of the GTP-bound form of Rab7A, and inhibits the Rab7A-mediated EGFR degradation. Silencing of CD44 in these cells causes an enhanced targeting of EGFR to the late endosome/lysosome compartments and leads to the strikingly faster EGFR degradation in response to EGF [103].

Connexins are components of gap junctions, which can also act as CAMs, mediating cell adhesion independently of gap junctions [104]. A knock-down of connexin32 using siRNA in C33-A cervical cancer cells results in a reduction in EGFR levels [105]. Inversely, induced expression of conenxin32 in HeLa cells leads to an increase in EGFR levels and activation of Erk1/2 and STAT3 signaling [105,106]. These effects are observed even in low-density cultures, indicating that they do not depend on the gap junction formation, which is blocked in these cultures [106]. In HepG2 and SMMC-7721 hepatocellular carcinoma cells, connexin 32 forms a complex with phosphorylated EGFR and c-Src [107]. Downregulation of connexin32 using siRNA in HepG2 cells, which endogenously express high levels of connexin32, results in a decrease in the mRNA and protein levels of EGFR and reduces the levels of activated EGFR, STAT3, and Erk1/2. In contrast, overexpression of connexin 32 in SMMC-7721 cells, which endogenously express low levels of connexin32, leads to an increase in the mRNA and protein levels of EGFR and increases levels of activated EGFR, STAT3, and Erk1/2. These effects can be reversed by overexpression of c-Src in HepG2 cells and knock-down of c-Src using siRNA in SMMC-7721 cells, indicating that connexin32 regulates expression and activity of EGFR via increasing expression of c-Src [107].

## 9. A Feedback Loop Between the CAM-Dependent EGFR Regulation and Cell Adhesion Modulation

Several reports suggest a feedback loop between the CAM-dependent EGFR regulation and cell adhesion modulation. In MCF10A cells, aggregation of E-cadherin using antibodies causes a reduction in number of focal adhesions. This effect is blocked by an EGFR inhibitor, indicating that changes in focal adhesions are induced by a ligand independent activation of EGFR by E-cadherin (Fedor-Chaiken et al., 2003). In A431 squamous carcinoma cells, EGF induces β1 integrin activation. This effect is reduced by silencing of T-cadherins and increased by T-cadherin overexpression. EGF-induces cell rounding and retraction from the substrate, and this effect is increased by silencing of T-cadherins and reduced by overexpression of T-cadherins, indicating that T-cadherins promote adhesion via the EGFR-dependent integrin activation [67]. In enterocytes, Desmoglein-2 induces c-Src dependent EGFR activation, leading to the strengthening of adhesion, while Desmoglein-2 suppresses cell proliferation by inhibiting the canonical EGFR signalling [70]. In wild-type human oral squamous carcinoma HSC3 cells, flat clathrin lattices at the plasma membrane accumulate β5 integrins and represent the sites of cell adhesion to the substrate. The size of these flat clathrin lattices increases in response to EGF, and this effect is blocked by pharmacological inhibition or knock-down of either EGFR or β5 integrins [83]. In colon cancer cells and mesenchymal stem cells, the extracellular domain of EpCAM shed from the cell surface via proteolytic cleavage mediated by ADAM17 and γ-secretase increases the activities of ADAM17 and γ-secretase by inducing their EGFR-dependent phosphorylation, thereby promoting further EpCAM shedding [97,98]. In MDA-MB-231 breast cancer cells, EGF induces an increase in levels of ICAM-1, while the EGFR inhibitor reduces ICAM-1 expression. EGFR regulates ICAM-1 expression by activating STAT3, which binds to the promoter of ICAM-1 [92].

## 10. Anti-Apoptotic Effects of the CAM-Dependent EGFR Activation

Cells of many types undergo apoptosis when deprived of adhesion to the appropriate extracellular matrix. However, adherent cells also undergo apoptosis in the absence of growth factors. EGFR-transfected NIH-3T3 cells enter apoptosis when kept in suspension for 24 h in serum-free medium but are rescued when plated on fibronectin even in the absence of growth factors. This integrin-dependent protection from programmed cell death in the absence of growth factors is abolished by inhibition of EGFR and EGFR-dependent PI3K signaling but is not affected by MAPK inhibitors [73].

The cadherin-mediated EGFR activation can also be anti-apoptotic. The E-cadherin-dependent activation of EGFR leads to an increase in expression of the anti-apoptotic protein Bcl-2 and promotes survival of cells in multicellular aggregates formed by squamous cell carcinoma cells (Shen and Kramer, 2004). The soluble extracellular domain of E-cadherin is released from the cell surface of Madin–Darby canine kidney (MDCK) epithelial cells via matrix metalloproteinase (MMP)-9 mediated cleavage of the full-length E-cadherin. This soluble extracellular domain of E-cadherin inhibits apoptosis in cysts formed by MDCK cells by activating EGFR and also inducing an increase in Bcl-2 expression (Patil et al., 2015). However, E-cadherin activation did not affect apoptosis rates in several other cell lines, including human mammary epithelial cells, human breast adenocarcinoma MCF-7, and human colon carcinoma HT29 cell lines [60].

## 11. The Role of the CAM-Dependent EGFR Regulation in Cell Proliferation

Cell adhesion is important for entry into the cell cycle, since EGF or serum do not trigger entry into G2/M phases in cells kept in suspension [73]. Integrin-induced activation of EGFR is sufficient to induce G1 cell cycle entry in CV1 cells, primary prostate epithelial cells, and keratinocytes [76] and triggers translocation of Akt to the nucleus and expression of early growth response gene Egr-1 [74]. In non-small cell carcinoma A549 cells, β1-integrin silencing inhibits EGFR-dependent cell proliferation even in the presence of serum [77], further indicating that integrins are required for cell proliferation. However, the integrin-induced ligand-independent activation of EGFR is not sufficient to induce DNA synthesis [76]. Accordingly, cells plated on fibronectin do not progress into G2/M phase unless EGF or serum is also present, indicating that integrin-dependent EGFR activation in response to fibronectin does not lead to a loss of requirement for growth factors to progress into the cell cycle [73].

The effects of cadherin-dependent EGFR regulation on cell proliferation are less clear and are probably cell-type dependent. In cultures of normal human breast epithelial cells, establishment of E-cadherin-mediated adhesion at cell-to-cell contacts coincides with inhibition of DNA synthesis and block of cell proliferation even in the presence of EGF. Disruption of the E-cadherin adhesion using an anti-cadherin antibody removes this block and results in DNA synthesis in response to EGF [59]. Induction of homophilic E-cadherin binding in human breast adenocarcinoma MCF-7 and the human colon cancer SW480 cells expressing E-cadherin by incubating cells with recombinant extracellular domain of E-cadherin results in inhibition of EGF-induced DNA synthesis [60]. In enterocytes, the loss of Desmoglein-2 results in an increase in EGFR-dependent cell proliferation [70]. Similarly, downregulation of Desmocollin-2 using siRNA results in an increase in EGFR-dependent proliferation of SK-CO15 cells [108]. However, overexpression of Desmoglein-2 in human squamous carcinoma A431 cells enhances EGFR activation and increases cell proliferation in a c-Src- and EGFR-dependent manner [71].

Several members of IgSF were implicated in promoting EGFR-dependent cell proliferation. Neuroglian is an IgSF CAM present in glia and neurons of the *Drosophila* nervous system and sharing high homology with mammalian L1CAM [109]. Neuroglian is also expressed in the intestinal stem cells and enteroblasts of the *Drosophila* midgut. Overexpression of neuroglian in the intestinal stem cells and enteroblasts increases intestinal stem cell proliferation, and this effect is suppressed by co-expression of the dominant negative EGFR, indicating that neuroglian induces intestinal stem cell proliferation by activating EGFR [110]. In the murine olfactory epithelium, proteolytic cleavage of the extracellular domain of neural glia-related CAM (NrCAM), a member of the L1CAM family, at the cell surface of olfactory horizontal basal cells produces a product that induces EGFR activation in these cells and promotes their proliferation and differentiation required for reconstruction of the olfactory epithelium following injury [111].

CAMs that do not belong to the major superfamilies also regulate proliferation of cells in an EGFR-dependent manner. The extracellular domain of EpCAM stimulates proliferation of colon cancer cells, and this effect is inhibited by an EGFR inhibitor [97]. Similarly, the extracellular domain of EpCAM induces mesenchymal stem cell proliferation and self-renewal, and these effects are also inhibited in cells treated with the EGFR inhibitor [98].

## 12. Conclusions

Extensive research over the last 30 years showed that the ligand-dependent and independent activity of EGFR is regulated by various CAMs belonging to different families (Table 1). CAMs can be broadly grouped depending on their effect on EGFR activity. CAMs belonging to the first group, which includes integrins, induce the ligand-independent activation of EGFR and potentiate its ligand-dependent activation. CAMs belonging to the second group, which includes E-cadherin and an IgSF member ALCAM, promote the ligand-independent EGFR activation and simultaneously block the ligand-dependent activation of EGFR, possibly by blocking the access of ligands for binding to EGFR. CAMs belonging to the third group, which includes Desmoglein-1 and an IgSF member NCAM180, downregulate EGFR activity. The effects of CAMs on EGFR activity may, however, also be cell-type-specific, possibly depending on the expression of EGFR. The CAM-dependent EGFR regulation plays a key role in the essential cellular processes, including cell proliferation and survival, which can be either enhanced or inhibited by targeting this molecular mechanism. Further research is, however, needed to identify additional CAMs that regulate EGFR and to fully understand the molecular details of the CAM-dependent EGFR regulation. Whether mutations in CAMs and EGFR contribute to the development of human diseases, including cancer, by affecting the CAM-dependent regulation of EGFR is an important question for further research. Altogether, this research is needed for the development of novel therapies targeting the CAM-dependent regulation of EGFR in human diseases.

## Figures and Tables

**Table 1:** Summary of the effects of CAMs on ligand-dependent and independent activation of EGFR.

Family	CAM	Ligand-Dependent EGFR Activation	Ligand-Independent EGFR Activation	Interaction with EGFR
Cadherins	E-cadherins	↓	↑	Yes
	T-cadherins	↓		
	Desmoglein-1	↓	↓	
	Desmoglein-2	↑	↑	Yes
	Desmocollin 2		↓	Yes
Integrins	β1 integrins	↑	↑	Yes
	Av integrins		↑	
	β3 integrins		↑	
	α1β1 integrins		↓	
	αvβ3 integrins		↑	Yes
	α5β1 integrins	↑	↑	Yes
IgSF	L1CAM		↑	Yes
	NrCAM		↑	Yes
	Neuroglian		↑	
	ALCAM	↓	↑	Yes
	ICAM2	↑		Yes
	CEACAM6	↑		Yes
	NCAM-180	↓		
Others	EpCAM		↑	
	Ninjurin2		↑	Yes
	CD44		↑	
	Connexin32	↑		Yes

## Data Availability

No new data were created or analyzed in this study. Data sharing is not applicable to this article.

## References

[1] Sheng L., Leshchyns’ka I., Sytnyk V. (2013). Cell adhesion and intracellular calcium signaling in neurons. Cell Commun. Signal..

[2] Kozlova I., Sah S., Keable R., Leshchyns’ka I., Janitz M., Sytnyk V. (2020). Cell adhesion molecules and protein synthesis regulation in neurons. Front. Mol. Neurosci..

[3] Keable R., Leshchyns’ka I., Sytnyk V. (2020). Trafficking and activity of glutamate and GABA receptors: Regulation by cell adhesion molecules. Neuroscientist.

[4] Leshchyns’ka I., Sytnyk V. (2016). Reciprocal interactions between cell adhesion molecules of the immunoglobulin superfamily and the cytoskeleton in neurons. Front. Cell Dev. Biol..

[5] Ono M., Kuwano M. (2006). Molecular mechanisms of epidermal growth factor receptor (EGFR) activation and response to gefitinib and other EGFR-targeting drugs. Clin. Cancer Res..

[6] Wee P., Wang Z. (2017). Epidermal growth factor receptor cell proliferation signaling pathways. Cancers.

[7] Gumbiner B.M. (1996). Cell adhesion: The molecular basis of tissue architecture and morphogenesis. Cell.

[8] Shapiro L., Love J., Colman D.R. (2007). Adhesion molecules in the nervous system: Structural insights into function and diversity. Annu. Rev. Neurosci..

[9] Yulis M., Kusters D.H.M., Nusrat A. (2018). Cadherins: Cellular adhesive molecules serving as signalling mediators. J. Physiol..

[10] Seong E., Yuan L., Arikkath J. (2015). Cadherins and catenins in dendrite and synapse morphogenesis. Cell Adh. Migr..

[11] Posy S., Shapiro L., Honig B. (2008). Sequence and structural determinants of strand swapping in cadherin domains: Do all cadherins bind through the same adhesive interface?. J. Mol. Biol..

[12] Boggon T.J., Murray J., Chappuis-Flament S., Wong E., Gumbiner B.M., Shapiro L. (2002). C-cadherin ectodomain structure and implications for cell adhesion mechanisms. Science.

[13] Rakshit S., Zhang Y., Manibog K., Shafraz O., Sivasankar S. (2012). Ideal, catch, and slip bonds in cadherin adhesion. Proc. Natl. Acad. Sci. USA.

[14] Sivasankar S., Xie B. (2023). Engineering the interactions of classical cadherin cell-cell adhesion proteins. J. Immunol..

[15] Chappuis-Flament S., Wong E., Hicks L.D., Kay C.M., Gumbiner B.M. (2001). Multiple cadherin extracellular repeats mediate homophilic binding and adhesion. J. Cell Biol..

[16] Harrison O.J., Jin X., Hong S., Bahna F., Ahlsen G., Brasch J., Wu Y., Vendome J., Felsovalyi K., Hampton C.M. (2011). The extracellular architecture of adherens junctions revealed by crystal structures of type I cadherins. Structure.

[17] Priest A.V., Shafraz O., Sivasankar S. (2017). Biophysical basis of cadherin mediated cell-cell adhesion. Exp. Cell Res..

[18] Thompson C.J., Vu V.H., Leckband D.E., Schwartz D.K. (2021). Cadherin cis and trans interactions are mutually cooperative. Proc. Natl. Acad. Sci. USA.

[19] Chitaev N.A., Leube R.E., Troyanovsky R.B., Eshkind L.G., Franke W.W., Troyanovsky S.M. (1996). The binding of plakoglobin to desmosomal cadherins: Patterns of binding sites and topogenic potential. J. Cell Biol..

[20] Kechagia J.Z., Ivaska J., Roca-Cusachs P. (2019). Integrins as biomechanical sensors of the microenvironment. Nat. Rev. Mol. Cell Biol..

[21] Barczyk M., Carracedo S., Gullberg D. (2010). Integrins. Cell Tissue Res..

[22] Campbell I.D., Humphries M.J. (2011). Integrin structure, activation, and interactions. Cold Spring Harb. Perspect. Biol..

[23] Kanchanawong P., Calderwood D.A. (2023). Organization, dynamics and mechanoregulation of integrin-mediated cell-ECM adhesions. Nat. Rev. Mol. Cell Biol..

[24] Tvaroska I., Kozmon S., Kona J. (2023). Molecular Modeling Insights into the Structure and Behavior of Integrins: A Review. Cells.

[25] Lu F., Zhu L., Bromberger T., Yang J., Yang Q., Liu J., Plow E.F., Moser M., Qin J. (2022). Mechanism of integrin activation by talin and its cooperation with kindlin. Nat. Commun..

[26] Zinn K., Ozkan E. (2017). Neural immunoglobulin superfamily interaction networks. Curr. Opin. Neurobiol..

[27] Tan R.P.A., Leshchyns’ka I., Sytnyk V. (2017). Glycosylphosphatidylinositol-anchored immunoglobulin superfamily cell adhesion molecules and their role in neuronal development and synapse regulation. Front. Mol. Neurosci..

[28] Sytnyk V., Leshchyns’ka I., Schachner M. (2017). Neural cell adhesion molecules of the immunoglobulin superfamily regulate synapse formation, maintenance, and function. Trends Neurosci..

[29] Pfundstein G., Nikonenko A.G., Sytnyk V. (2022). Amyloid precursor protein (APP) and amyloid beta (Abeta) interact with cell adhesion molecules: Implications in Alzheimer’s disease and normal physiology. Front. Cell Dev. Biol..

[30] Wojtowicz W.M., Vielmetter J., Fernandes R.A., Siepe D.H., Eastman C.L., Chisholm G.B., Cox S., Klock H., Anderson P.W., Rue S.M. (2020). A human IgSF cell-surface interactome reveals a complex network of protein-protein interactions. Cell.

[31] Ranaivoson F.M., Turk L.S., Ozgul S., Kakehi S., von Daake S., Lopez N., Trobiani L., De Jaco A., Denissova N., Demeler B. (2019). A proteomic screen of neuronal cell-surface molecules reveals IgLONs as structurally conserved interaction modules at the synapse. Structure.

[32] Duncan B.W., Murphy K.E., Maness P.F. (2021). Molecular mechanisms of L1 and NCAM adhesion molecules in synaptic pruning, plasticity, and stabilization. Front. Cell Dev. Biol..

[33] Schneider M.R., Wolf E. (2009). The epidermal growth factor receptor ligands at a glance. J. Cell Physiol..

[34] Schultz D.F., Billadeau D.D., Jois S.D. (2023). EGFR trafficking: Effect of dimerization, dynamics, and mutation. Front. Oncol..

[35] Lemmon M.A., Schlessinger J., Ferguson K.M. (2014). The EGFR family: Not so prototypical receptor tyrosine kinases. Cold Spring Harb. Perspect. Biol..

[36] Ferguson K.M., Berger M.B., Mendrola J.M., Cho H.S., Leahy D.J., Lemmon M.A. (2003). EGF activates its receptor by removing interactions that autoinhibit ectodomain dimerization. Mol. Cell.

[37] Kovacs E., Zorn J.A., Huang Y., Barros T., Kuriyan J. (2015). A structural perspective on the regulation of the epidermal growth factor receptor. Annu. Rev. Biochem..

[38] Ferguson K.M. (2008). Structure-based view of epidermal growth factor receptor regulation. Annu. Rev. Biophys..

[39] Zhang X., Gureasko J., Shen K., Cole P.A., Kuriyan J. (2006). An allosteric mechanism for activation of the kinase domain of epidermal growth factor receptor. Cell.

[40] Schulze W.X., Deng L., Mann M. (2005). Phosphotyrosine interactome of the ErbB-receptor kinase family. Mol. Syst. Biol..

[41] Olsen J.V., Blagoev B., Gnad F., Macek B., Kumar C., Mortensen P., Mann M. (2006). Global, in vivo, and site-specific phosphorylation dynamics in signaling networks. Cell.

[42] Knudsen S.L., Mac A.S., Henriksen L., van Deurs B., Grovdal L.M. (2014). EGFR signaling patterns are regulated by its different ligands. Growth Factors.

[43] Wang Q., Villeneuve G., Wang Z. (2005). Control of epidermal growth factor receptor endocytosis by receptor dimerization, rather than receptor kinase activation. EMBO Rep..

[44] Sousa L.P., Lax I., Shen H., Ferguson S.M., De Camilli P., Schlessinger J. (2012). Suppression of EGFR endocytosis by dynamin depletion reveals that EGFR signaling occurs primarily at the plasma membrane. Proc. Natl. Acad. Sci. USA.

[45] Burke P., Schooler K., Wiley H.S. (2001). Regulation of epidermal growth factor receptor signaling by endocytosis and intracellular trafficking. Mol. Biol. Cell.

[46] Huang F., Khvorova A., Marshall W., Sorkin A. (2004). Analysis of clathrin-mediated endocytosis of epidermal growth factor receptor by RNA interference. J. Biol. Chem..

[47] Goh L.K., Huang F., Kim W., Gygi S., Sorkin A. (2010). Multiple mechanisms collectively regulate clathrin-mediated endocytosis of the epidermal growth factor receptor. J. Cell Biol..

[48] Alfonzo-Mendez M.A., Strub M.P., Taraska J.W. (2024). Spatial and signaling overlap of growth factor receptor systems at clathrin-coated sites. Mol. Biol. Cell.

[49] Sigismund S., Woelk T., Puri C., Maspero E., Tacchetti C., Transidico P., Di Fiore P.P., Polo S. (2005). Clathrin-independent endocytosis of ubiquitinated cargos. Proc. Natl. Acad. Sci. USA.

[50] Sigismund S., Argenzio E., Tosoni D., Cavallaro E., Polo S., Di Fiore P.P. (2008). Clathrin-mediated internalization is essential for sustained EGFR signaling but dispensable for degradation. Dev. Cell.

[51] Wilde A., Beattie E.C., Lem L., Riethof D.A., Liu S.H., Mobley W.C., Soriano P., Brodsky F.M. (1999). EGF receptor signaling stimulates SRC kinase phosphorylation of clathrin, influencing clathrin redistribution and EGF uptake. Cell.

[52] Orth J.D., Krueger E.W., Weller S.G., McNiven M.A. (2006). A novel endocytic mechanism of epidermal growth factor receptor sequestration and internalization. Cancer Res..

[53] Bakker J., Spits M., Neefjes J., Berlin I. (2017). The EGFR odyssey—from activation to destruction in space and time. J. Cell Sci..

[54] Goh L.K., Sorkin A. (2013). Endocytosis of receptor tyrosine kinases. Cold Spring Harb. Perspect. Biol..

[55] Roepstorff K., Grandal M.V., Henriksen L., Knudsen S.L., Lerdrup M., Grovdal L., Willumsen B.M., van Deurs B. (2009). Differential effects of EGFR ligands on endocytic sorting of the receptor. Traffic.

[56] Huang F., Goh L.K., Sorkin A. (2007). EGF receptor ubiquitination is not necessary for its internalization. Proc. Natl. Acad. Sci. USA.

[57] Eden E.R., Huang F., Sorkin A., Futter C.E. (2012). The role of EGF receptor ubiquitination in regulating its intracellular traffic. Traffic.

[58] Alwan H.A., van Zoelen E.J., van Leeuwen J.E. (2003). Ligand-induced lysosomal epidermal growth factor receptor (EGFR) degradation is preceded by proteasome-dependent EGFR de-ubiquitination. J. Biol. Chem..

[59] Takahashi K., Suzuki K. (1996). Density-dependent inhibition of growth involves prevention of EGF receptor activation by E-cadherin-mediated cell-cell adhesion. Exp. Cell Res..

[60] Perrais M., Chen X., Perez-Moreno M., Gumbiner B.M. (2007). E-cadherin homophilic ligation inhibits cell growth and epidermal growth factor receptor signaling independently of other cell interactions. Mol. Biol. Cell.

[61] Pece S., Gutkind J.S. (2000). Signaling from E-cadherins to the MAPK pathway by the recruitment and activation of epidermal growth factor receptors upon cell-cell contact formation. J. Biol. Chem..

[62] Shen X., Kramer R.H. (2004). Adhesion-mediated squamous cell carcinoma survival through ligand-independent activation of epidermal growth factor receptor. Am. J. Pathol..

[63] Fedor-Chaiken M., Hein P.W., Stewart J.C., Brackenbury R., Kinch M.S. (2003). E-cadherin binding modulates EGF receptor activation. Cell Commun. Adhes..

[64] Russo G.C., Crawford A.J., Clark D., Cui J., Carney R., Karl M.N., Su B., Starich B., Lih T.S., Kamat P. (2024). E-cadherin interacts with EGFR resulting in hyper-activation of ERK in multiple models of breast cancer. Oncogene.

[65] Fu C., Dilasser F., Lin S.Z., Karnat M., Arora A., Rajendiran H., Ong H.T., Mui Hoon Brenda N., Phow S.W., Hirashima T. (2024). Regulation of intercellular viscosity by E-cadherin-dependent phosphorylation of EGFR in collective cell migration. Proc. Natl. Acad. Sci. USA.

[66] Patil P.U., D’Ambrosio J., Inge L.J., Mason R.W., Rajasekaran A.K. (2015). Carcinoma cells induce lumen filling and EMT in epithelial cells through soluble E-cadherin-mediated activation of EGFR. J. Cell Sci..

[67] Kyriakakis E., Maslova K., Philippova M., Pfaff D., Joshi M.B., Buechner S.A., Erne P., Resink T.J. (2012). T-Cadherin is an auxiliary negative regulator of EGFR pathway activity in cutaneous squamous cell carcinoma: Impact on cell motility. J. Invest. Dermatol..

[68] Getsios S., Simpson C.L., Kojima S., Harmon R., Sheu L.J., Dusek R.L., Cornwell M., Green K.J. (2009). Desmoglein 1-dependent suppression of EGFR signaling promotes epidermal differentiation and morphogenesis. J. Cell Biol..

[69] Valenzuela-Iglesias A., Burks H.E., Arnette C.R., Yalamanchili A., Nekrasova O., Godsel L.M., Green K.J. (2019). Desmoglein 1 regulates invadopodia by suppressing EGFR/Erk signaling in an erbin-dependent manner. Mol. Cancer Res..

[70] Ungewiss H., Rotzer V., Meir M., Fey C., Diefenbacher M., Schlegel N., Waschke J. (2018). Dsg2 via Src-mediated transactivation shapes EGFR signaling towards cell adhesion. Cell Mol. Life Sci..

[71] Overmiller A.M., McGuinn K.P., Roberts B.J., Cooper F., Brennan-Crispi D.M., Deguchi T., Peltonen S., Wahl J.K., Mahoney M.G. (2016). c-Src/Cav1-dependent activation of the EGFR by Dsg2. Oncotarget.

[72] Kamekura R., Kolegraff K.N., Nava P., Hilgarth R.S., Feng M., Parkos C.A., Nusrat A. (2014). Loss of the desmosomal cadherin desmoglein-2 suppresses colon cancer cell proliferation through EGFR signaling. Oncogene.

[73] Moro L., Venturino M., Bozzo C., Silengo L., Altruda F., Beguinot L., Tarone G., Defilippi P. (1998). Integrins induce activation of EGF receptor: Role in MAP kinase induction and adhesion-dependent cell survival. EMBO J..

[74] Cabodi S., Morello V., Masi A., Cicchi R., Broggio C., Distefano P., Brunelli E., Silengo L., Pavone F., Arcangeli A. (2009). Convergence of integrins and EGF receptor signaling via PI3K/Akt/FoxO pathway in early gene Egr-1 expression. J. Cell Physiol..

[75] Wang X.Q., Sun P., Paller A.S. (2003). Ganglioside GM3 blocks the activation of epidermal growth factor receptor induced by integrin at specific tyrosine sites. J. Biol. Chem..

[76] Bill H.M., Knudsen B., Moores S.L., Muthuswamy S.K., Rao V.R., Brugge J.S., Miranti C.K. (2004). Epidermal growth factor receptor-dependent regulation of integrin-mediated signaling and cell cycle entry in epithelial cells. Mol. Cell Biol..

[77] Morello V., Cabodi S., Sigismund S., Camacho-Leal M.P., Repetto D., Volante M., Papotti M., Turco E., Defilippi P. (2011). beta1 integrin controls EGFR signaling and tumorigenic properties of lung cancer cells. Oncogene.

[78] Mattila E., Pellinen T., Nevo J., Vuoriluoto K., Arjonen A., Ivaska J. (2005). Negative regulation of EGFR signalling through integrin-alpha1beta1-mediated activation of protein tyrosine phosphatase TCPTP. Nat. Cell Biol..

[79] Roovers K., Davey G., Zhu X., Bottazzi M.E., Assoian R.K. (1999). Alpha5beta1 integrin controls cyclin D1 expression by sustaining mitogen-activated protein kinase activity in growth factor-treated cells. Mol. Biol. Cell.

[80] Miyamoto S., Teramoto H., Gutkind J.S., Yamada K.M. (1996). Integrins can collaborate with growth factors for phosphorylation of receptor tyrosine kinases and MAP kinase activation: Roles of integrin aggregation and occupancy of receptors. J. Cell Biol..

[81] Moro L., Dolce L., Cabodi S., Bergatto E., Boeri Erba E., Smeriglio M., Turco E., Retta S.F., Giuffrida M.G., Venturino M. (2002). Integrin-induced epidermal growth factor (EGF) receptor activation requires c-Src and p130Cas and leads to phosphorylation of specific EGF receptor tyrosines. J. Biol. Chem..

[82] Caswell P.T., Chan M., Lindsay A.J., McCaffrey M.W., Boettiger D., Norman J.C. (2008). Rab-coupling protein coordinates recycling of alpha5beta1 integrin and EGFR1 to promote cell migration in 3D microenvironments. J. Cell Biol..

[83] Alfonzo-Mendez M.A., Sochacki K.A., Strub M.P., Taraska J.W. (2022). Dual clathrin and integrin signaling systems regulate growth factor receptor activation. Nat. Commun..

[84] Mukoyama Y., Utani A., Matsui S., Zhou S., Miyachi Y., Matsuyoshi N. (2007). T-cadherin enhances cell-matrix adhesiveness by regulating beta1 integrin trafficking in cutaneous squamous carcinoma cells. Genes Cells.

[85] Islam R., Kristiansen L.V., Romani S., Garcia-Alonso L., Hortsch M. (2004). Activation of EGF receptor kinase by L1-mediated homophilic cell interactions. Mol. Biol. Cell.

[86] Nagaraj K., Kristiansen L.V., Skrzynski A., Castiella C., Garcia-Alonso L., Hortsch M. (2009). Pathogenic human L1-CAM mutations reduce the adhesion-dependent activation of EGFR. Hum. Mol. Genet..

[87] Donier E., Gomez-Sanchez J.A., Grijota-Martinez C., Lakoma J., Baars S., Garcia-Alonso L., Cabedo H. (2012). L1CAM binds ErbB receptors through Ig-like domains coupling cell adhesion and neuregulin signalling. PLoS ONE.

[88] Zhang Y., Sui F., Ma J., Ren X., Guan H., Yang Q., Shi J., Ji M., Shi B., Sun Y. (2017). Positive feedback loops between NrCAM and major signaling pathways contribute to thyroid tumorigenesis. J. Clin. Endocrinol. Metab..

[89] Garcia-Alonso L., Romani S., Jimenez F. (2000). The EGF and FGF receptors mediate neuroglian function to control growth cone decisions during sensory axon guidance in Drosophila. Neuron.

[90] Chen X., Liang R., Lin H., Chen K., Chen L., Tian G., Zhu X. (2021). CD166 promotes cancer stem cell-like phenotype via the EGFR/ERK1/2 pathway in the nasopharyngeal carcinoma cell line CNE-2R. Life Sci..

[91] Luo H., Zhang D., Wang F., Wang Q., Wu Y., Gou M., Hu Y., Zhang W., Huang J., Gong Y. (2021). ALCAM-EGFR interaction regulates myelomagenesis. Blood Adv..

[92] Kang J.H., Uddin N., Kim S., Zhao Y., Yoo K.C., Kim M.J., Hong S.A., Bae S., Lee J.Y., Shin I. (2024). Tumor-intrinsic role of ICAM-1 in driving metastatic progression of triple-negative breast cancer through direct interaction with EGFR. Mol. Cancer.

[93] Chiang W.F., Cheng T.M., Chang C.C., Pan S.H., Changou C.A., Chang T.H., Lee K.H., Wu S.Y., Chen Y.F., Chuang K.H. (2018). Carcinoembryonic antigen-related cell adhesion molecule 6 (CEACAM6) promotes EGF receptor signaling of oral squamous cell carcinoma metastasis via the complex N-glycosylation. Oncogene.

[94] Povlsen G.K., Berezin V., Bock E. (2008). Neural cell adhesion molecule-180-mediated homophilic binding induces epidermal growth factor receptor (EGFR) down-regulation and uncouples the inhibitory function of EGFR in neurite outgrowth. J. Neurochem..

[95] Kristiansen L.V., Velasquez E., Romani S., Baars S., Berezin V., Bock E., Hortsch M., Garcia-Alonso L. (2005). Genetic analysis of an overlapping functional requirement for L1- and NCAM-type proteins during sensory axon guidance in Drosophila. Mol. Cell Neurosci..

[96] Litvinov S.V., Velders M.P., Bakker H.A., Fleuren G.J., Warnaar S.O. (1994). Ep-CAM: A human epithelial antigen is a homophilic cell-cell adhesion molecule. J. Cell Biol..

[97] Liang K.H., Tso H.C., Hung S.H., Kuan I.-I., Lai J.K., Ke F.Y., Chuang Y.T., Liu I.J., Wang Y.P., Chen R.H. (2018). Extracellular domain of EpCAM enhances tumor progression through EGFR signaling in colon cancer cells. Cancer Lett..

[98] Kuan I.-I., Lee C.C., Chen C.H., Lu J., Kuo Y.S., Wu H.C. (2019). The extracellular domain of epithelial cell adhesion molecule (EpCAM) enhances multipotency of mesenchymal stem cells through EGFR-LIN28-LET7 signaling. J. Biol. Chem..

[99] Araki T., Milbrandt J. (2000). Ninjurin2, a novel homophilic adhesion molecule, is expressed in mature sensory and enteric neurons and promotes neurite outgrowth. J. Neurosci..

[100] Zhou L.N., Li P., Cai S., Li G., Liu F. (2019). Ninjurin2 overexpression promotes glioma cell growth. Aging.

[101] Li G., Zhou L.N., Yang H., He X., Duan Y., Wu F. (2019). Ninjurin 2 overexpression promotes human colorectal cancer cell growth in vitro and in vivo. Aging.

[102] Goodison S., Urquidi V., Tarin D. (1999). CD44 cell adhesion molecules. Mol. Pathol..

[103] Wang W., Zhang H., Liu S., Kim C.K., Xu Y., Hurley L.A., Nishikawa R., Nagane M., Hu B., Stegh A.H. (2017). Internalized CD44s splice isoform attenuates EGFR degradation by targeting Rab7A. Proc. Natl. Acad. Sci. USA.

[104] Cotrina M.L., Lin J.H., Nedergaard M. (2008). Adhesive properties of connexin hemichannels. Glia.

[105] Zhao Y., Lai Y., Ge H., Guo Y., Feng X., Song J., Wang Q., Fan L., Peng Y., Cao M. (2017). Non-junctional Cx32 mediates anti-apoptotic and pro-tumor effects via epidermal growth factor receptor in human cervical cancer cells. Cell Death Dis..

[106] Lai Y., Tao L., Zhao Y., Zhang X., Sun X., Wang Q., Xu C. (2017). Cx32 inhibits TNFalpha-induced extrinsic apoptosis with and without EGFR suppression. Oncol. Rep..

[107] Xiang Y., Wang Q., Guo Y., Ge H., Fu Y., Wang X., Tao L. (2019). Cx32 exerts anti-apoptotic and pro-tumor effects via the epidermal growth factor receptor pathway in hepatocellular carcinoma. J. Exp. Clin. Cancer Res..

[108] Kolegraff K., Nava P., Helms M.N., Parkos C.A., Nusrat A. (2011). Loss of desmocollin-2 confers a tumorigenic phenotype to colonic epithelial cells through activation of Akt/beta-catenin signaling. Mol. Biol. Cell.

[109] Bieber A.J., Snow P.M., Hortsch M., Patel N.H., Jacobs J.R., Traquina Z.R., Schilling J., Goodman C.S. (1989). Drosophila neuroglian: A member of the immunoglobulin superfamily with extensive homology to the vertebrate neural adhesion molecule L1. Cell.

[110] Resnik-Docampo M., Cunningham K.M., Ruvalcaba S.M., Choi C., Sauer V., Jones D.L. (2021). Neuroglian regulates Drosophila intestinal stem cell proliferation through enhanced signaling via the epidermal growth factor receptor. Stem Cell Rep..

[111] Chen Z.H., Luo X.C., Yu C.R., Huang L. (2020). Matrix metalloprotease-mediated cleavage of neural glial-related cell adhesion molecules activates quiescent olfactory stem cells via EGFR. Mol. Cell Neurosci..

